# Supplementary material for: Regional complexity in enteric neuron wiring reflects diversity of motility patterns in the mouse large intestine
Source: eLife. 2019 Feb 12;8:e42914. doi: 10.7554/eLife.42914 (PMC6391068; doi:10.7554/eLife.42914)
Supplement: Source code 2. [file elife-42914-code2.pdf]

Installation and usage guide for the Ca-image Analysis package in Igor

Pieter Vanden Berghe, KU Leuven

© 2005-2019

With contributions by: Y. Kazwiny, M. Martens, W. Boesmans

Installation guide and terms of use

- Download Igor8 from www.wavemetrics.com
    - A demo version can be used for up to a month
    - A purchased license key should be entered when installing the full version
- Implement the custom written routines from prof. Pieter Vanden Berghe
    - Unzip from Vanden_Berghe_CaImaging_Analysis and
    - paste the all folders in the user\documents folder:

  …\WaveMetrics\Igor Pro 8 User Files\Igor procedures

- Do regularly check www.targid.eu > LENS for updates and upgrades
- Download and use of this package implies the explicit agreement to
    - Only use the software for academic research (excluding contract research)
    - Acknowledging the use of this package in the main Methods section of any type of publication, as follows: *"Ca imaging analysis was performed using Igor based on the custom routines written by P. Vanden Berghe (KU Leuven)"*
    - refer to the following two papers: *Boesmans et al. Frontiers 2013* (doi: 10.3389/fncel.2013.00183) and *Li et al. eLife 2019*
    - Not copy and change the code without explicit consent of the authors

User guide

Part I: Loading image stack and image stack operations

Open and upload image stack

- Open Igor software
- Go to > Load images > Load *.Tiff image in the Ca-Image Analysis P.VdB menu.

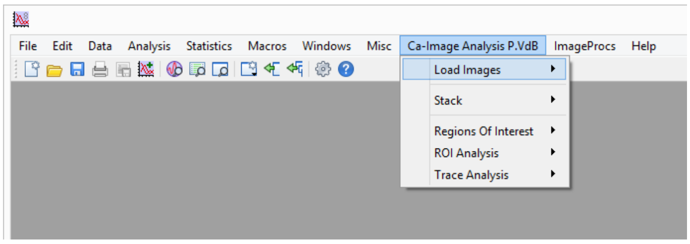

- Prompt for frame to frame interval (in ms)
- Option to rename the stack, short names are recommended, removing *.tiff extensions as well.
- Names should be between " "

- The package is not multicolor aware. Grayscale and LUT images should be opened as “No=0” RGB color images (“Yes=1”) will be split in three channels.

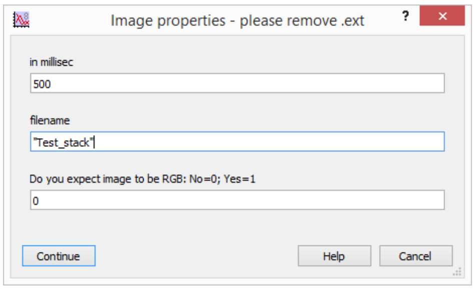

- Other types of formats should be prepared in other software packages to fit the required tiff (or raw) format.
- !! Calibrations are stripped from the original image format. All analysis is performed in pixels and frames !!
- Keeping track of pixel dimensions and frame intervals is the responsibility of the end user !

Browse image stack - display

- LUT and color range can be adapted from Display color panel.
- Do move the sliders. If left untouched the scaling will be automatically min/max, which might be confusing.

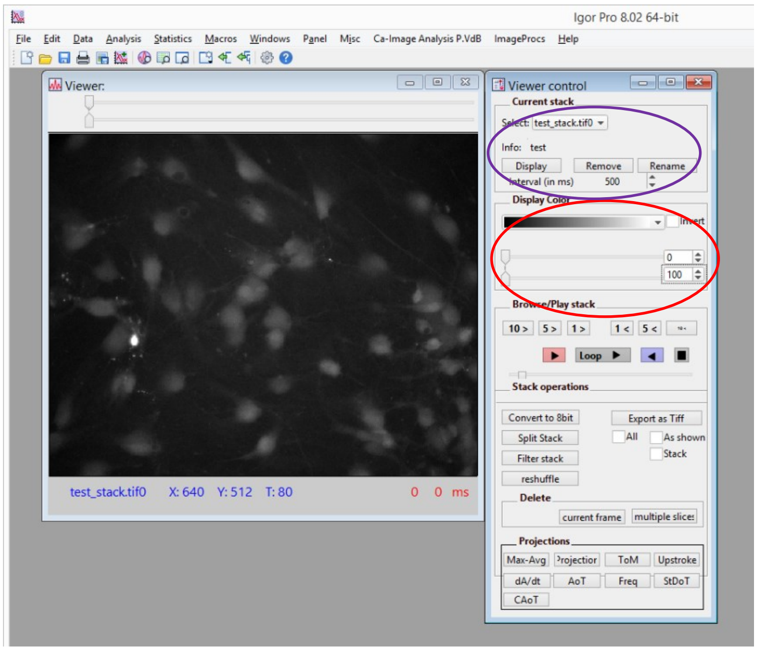

- Multiple imagestacks can be uploaded, removed, renamed… until RAM is (half)full.
- Browse in time, via the slider on top or the buttons in the Browse/Play panel.
- Loop will play the movie until mouse click elsewhere. The slider below determines the replay speed.

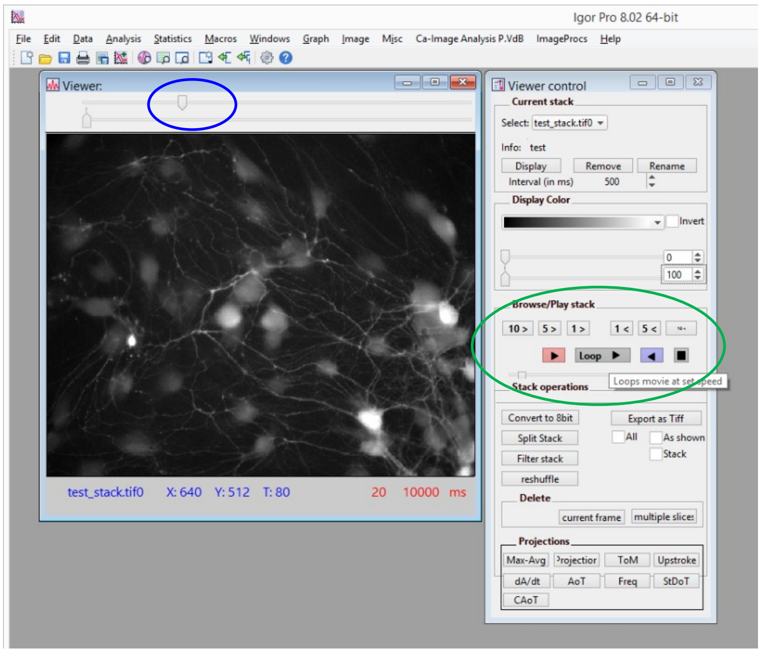

Stack operations

- Includes conversion to 8bit
- Splitting, filtering, exporting (as individual frames, as shown – depends on the display sliders, or as a multilayered tiff = Stack)

Projection

- Projection: can generate a Min, Max or Avg projection

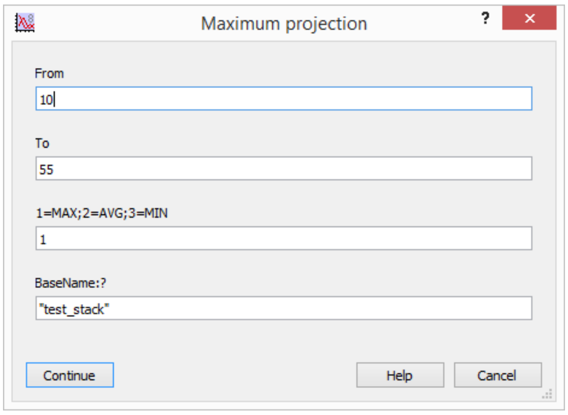

- Max-Avg: a maximum projection minus an average
- An Activity over Time image, or Time of maximum images: here every pixel of the image is converted to the maximum amplitude during a timewindow (AoT) or to the timepoint when that maximum occurred (ToM) (see Boesmans et al. Frontiers Cell.Neuroscience 2013).
- A dialogues is prompted to indicate the timewindow of interest, as well as (for AoT and ToM) a threshold (X times noise+ baseline) above which the signal is considered relevant, below which the signal is set to zero (see Boesmans et al. Frontiers Cell.Neuroscience 2013).

Part II: Defining Regions of interest + extraction of fluorescence intensity

Regions of interest

- Draw rectangular regions of interest > left mouse button click > marching ant selection.
- Hit F2 button, or activate ROI panel via the menu (Ca-image Analysis P.VdB > ROI)

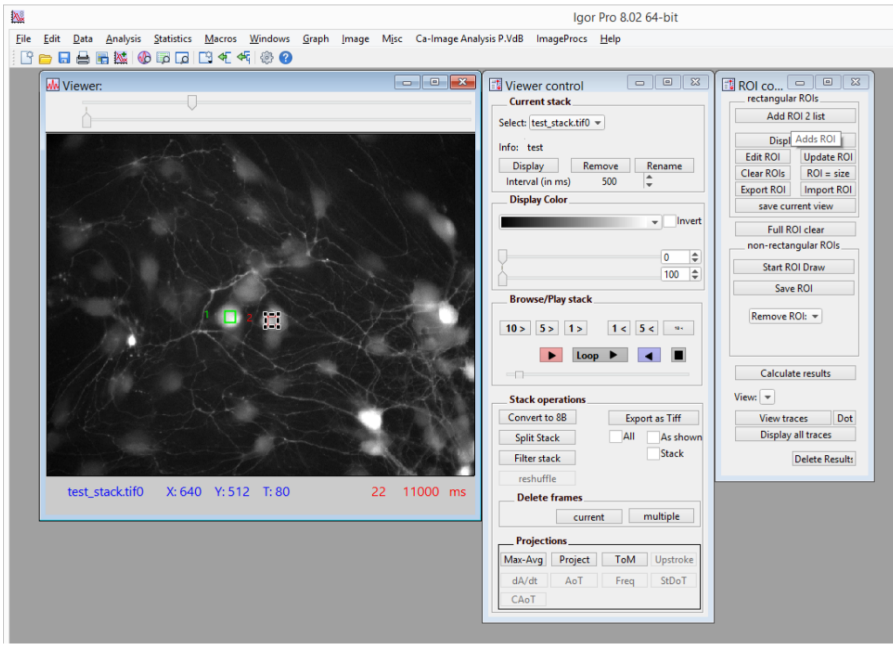

- Draw as many ROI as you like, the first 30 will have a unique color. Regions numbered 31 and higher will be the same color.
- ROI can be manipulated by clicking the “Edit ROI button”, editing should always be terminated by clicking “Update ROI”

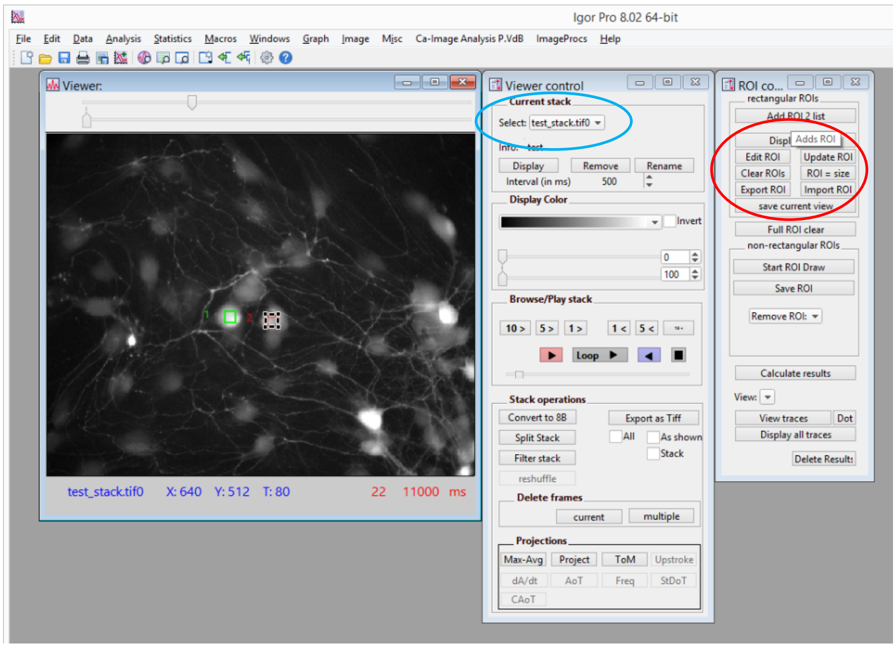

- ROI can be exported as a simple text file and imported again.
- Only one set of active ROI’s per session.

- ROI's are not restricted to one imagestack but are propagated over any imagestack that is activated by the "Select: ….." menu.
- Other shape ROI can be drawn using "non-rectangular ROIs"
- "Calculate results" computes the fluorescence traces over time for the imagestack as activated in "Select: ….." averaged for each ROI.
- View traces calls a viewer, which shows color matched traces, with a navigator slider on the left hand side of the viewer.
- View: when multiple results have been generated, here one can select the one to be viewed.
- Display all traces: Generates a graph with all traces as well as a table with all values, which can be copied to other programs (ctrl A to select all, ctrl C to copy).

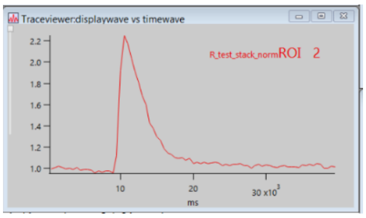

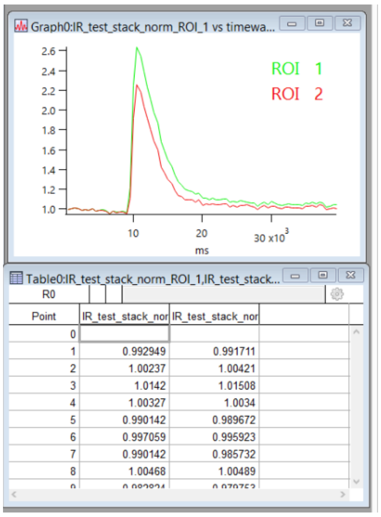

Part III: Analysis of the Ca transients

- Call trace analysis panel (Analyze traces) from top menu.

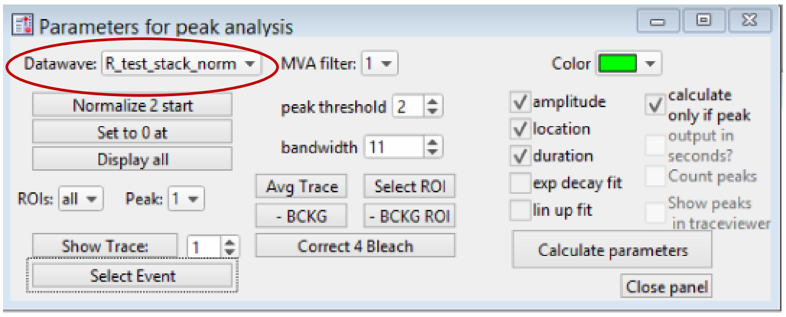

- Select the trace you want to work with (via Datawave: dropdown)
- "Normalize 2 start": allows to normalize the traces to the start (or any other time) of the recording. The selected frame + X frames is used to calculated the value to normalize to.
- The new results name will be: "*original*_norm"

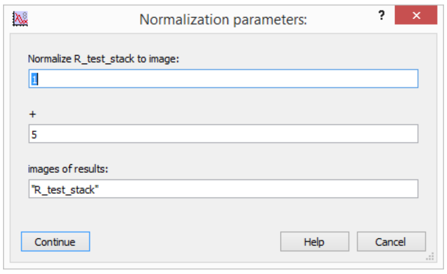

- Similarly "Set to 0 at" allows to subtract the values at a certain timepoint. Be careful to perform this operation on the correct results ("R_ ") as in Datawave dropdown.
- "Display all" shows all traces of the selection in one graph

- MVA filter: is a moving average filter that will be applied just before your calculations. 1 = no filter. Any other uneven number will smooth the traces. Apply only in case of high noise.
- Peak threshold is a factor that the noise (standard deviation of the signal at rest) will be multiplied by and added to the baseline (average of that signal) to determine the cutoff for peak detection.
- Bandwidth: is the number of frames that will be used at the start of the interval selected around the peak.
- Select event: generates a graph of the trace (selected in “show trace”) and waits for user interaction to move the cursors (red circle: left) before the start (allow some baseline) and after the end of the transient (black square).

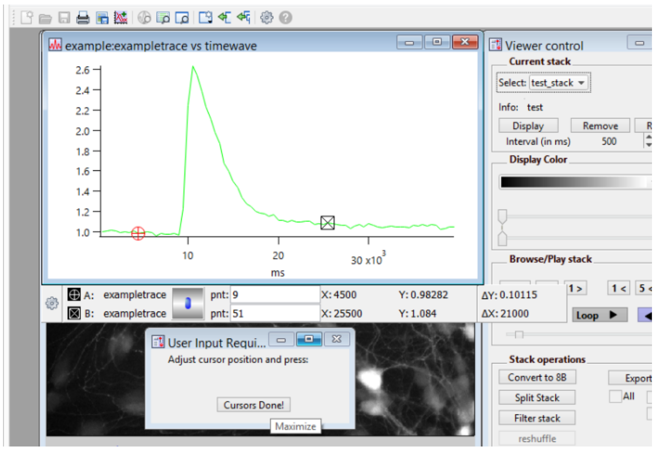

- NOTE:
  - this package was originally designed to analyze transients that were induced (electrically or pharmacologically) at clearly defined timepoints.
  - This window of selection (as indicated by the cursors) will be maintained for all traces.
  - Therefore spontaneously occurring transients that are not phase locked should not be analyzed with this package. Routines to address extra complexity are being developed.
- The Avg Trace and Select Roi buttons, generate average traces +/- SEM of all or selected traces. Generated graphs can be copied to PPT or other presentation software packages.
- -BCKG or –BCKG ROI: subtracts a fixed value or the values of one (bckg) region from all traces as selected in the Datawave dropdown

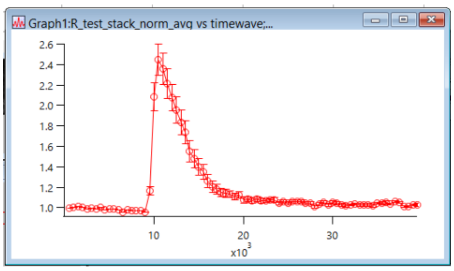

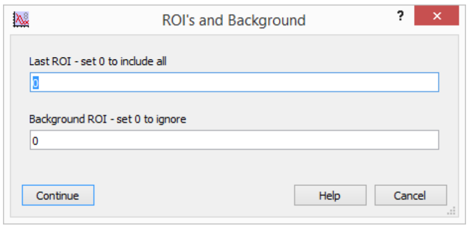

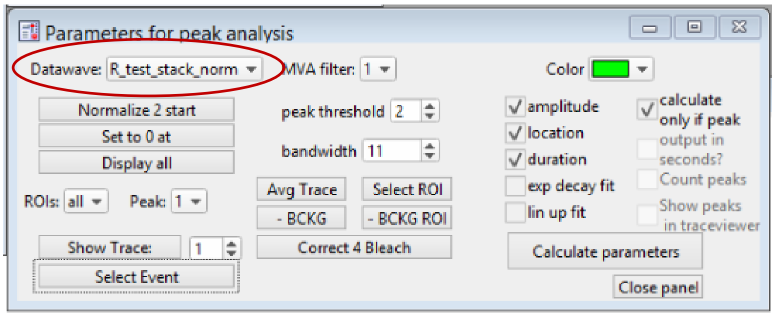

- The Correct 4 Bleach button can be used to correct the traces (as selected in the Datawave dropdown) for bleaching, provided that a mono-exponential fit is made through one trace that perfectly represents the bleaching. It is far more important to prevent bleaching by adjusting dye concentration and imaging parameters during the experiment. The physiological effects of bleaching obviously cannot be corrected offline.
- Peak amplitude, location (at maximum) and duration (10, 50, 90% duration see Vanden Berghe P et al. NGM 2002, PMID: 11874555) are calculated when a peak (that rises above threshold – "calculate only if peak") is detected.
- "exp decay fit" calls an interactive fitting module that prompts the user to agree with the quality of the fit. This slows the analysis substantially.
- "lin up fit" fits a line through the upstroke (from start to max)
- "Calculate parameters" computes the activated parameters per ROI (all or 1, 2 ,…) for each of the peaks marked (usually only one)

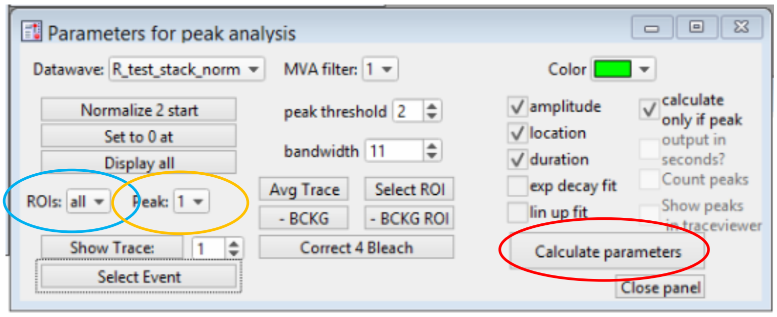

- A results table is generated, containing all parameters: values before the start (that is before normalization), the average before the peak, whether it was a responder or not, the maximum amplitude, the frame at which the peak started etc.

Table1:Peak_1_R_test_stack_norm.ld

R0 Label

Row	Peak_1_R_test_st x	Peak_1_R_test_st y	Peak_1_R_test_st interval_ms	Peak_1_R_test_st startB4norm	Peak_1_R_test_st avgB4peak	Peak_1_R_test_st responder	Peak_1_R_test_st peak_max	Peak_1_R_test_st peak_start	Peak_1_R_test_st peak_loc	Peak_1_R_test_st peakdur10	Peak_1_R_test_st peakdur50	Peak_1_R_test_st peakdur90	Peak_1_R_test_st fitpeak_max
0			500	0									
1				32.5991	1.10391	1	2.6358	19	21	4	9	15	2.6358
2				30.6625	1.06297	1	2.25275	19	21	4	8	14	2.25275
3													

- This table can be copied and used for further analysis in Igor or in any other spreadsheet type of program.

Please check www.targid.eu > LENS regularly for updates and upgrades. Contact information is also available via this site.
