# Supplementary material for: Regional complexity in enteric neuron wiring reflects diversity of motility patterns in the mouse large intestine
Source: eLife. 2019 Feb 12;8:e42914. doi: 10.7554/eLife.42914 (PMC6391068; doi:10.7554/eLife.42914)
Supplement: Transparent reporting form [file elife-42914-transrepform.docx]

***eLife’s* transparent reporting form**

We encourage authors to provide detailed information *within their submission* to facilitate the interpretation and replication of experiments. Authors can upload supporting documentation to indicate the use of appropriate reporting guidelines for health-related research (see [EQUATOR Network](http://www.equator-network.org/%20)), life science research (see the [BioSharing Information Resource](https://biosharing.org/)), or the [ARRIVE guidelines](http://www.plosbiology.org/article/info:doi/10.1371/journal.pbio.1000412) for reporting work involving animal research. Where applicable, authors should refer to any relevant reporting standards documents in this form.

If you have any questions, please consult our Journal Policies and/or contact us: [editorial@elifesciences.org](mailto:editorial@elifesciences.org).

**Sample-size estimation**

- You should state whether an appropriate sample size was computed when the study was being designed
- You should state the statistical method of sample size computation and any required assumptions
- If no explicit power analysis was used, you should describe how you decided what sample (replicate) size (number) to use

Please outline where this information can be found within the submission (e.g., sections or figure legends), or explain why this information doesn’t apply to your submission:

We did not determine a formal sample size calculation as this was the first time this type of experiment (low magnification Ca imaging combined with spatial mapping) was performed. Given the distribution of the responses (close to perfectly Gaussian in the controls) as well as the distribution of the locations, which resembled (arrows in fig 5) the organization of ganglia we are confident that our sample sizes were sufficiently large to draw sound conclusions. The numbers of recordings and repeats were performed in line with previous studies of our group in which we investigated Ca responses in enteric neurons using higher magnification.

All these experiments used mice and experimental procedures were approved by the animal ethics committee of KU Leuven (project # 021/2015).

**Replicates**

- You should report how often each experiment was performed
- You should include a definition of biological versus technical replication
- The data obtained should be provided and sufficient information should be provided to indicate the number of independent biological and/or technical replicates
- If you encountered any outliers, you should describe how these were handled
- Criteria for exclusion/inclusion of data should be clearly stated
- High-throughput sequence data should be uploaded before submission, with a private link for reviewers provided (these are available from both GEO and ArrayExpress)

Please outline where this information can be found within the submission (e.g., sections or figure legends), or explain why this information doesn’t apply to your submission:

All information about the numbers of neurons that were used to calculate the average responses (fig 1), the number of stimulation pairs (fig 3 and 4) to calculate the response ratio of each neuron as well as the distribution of those response ratios (histograms), locations were given in the text or figure legends. Also the number of animals (N) was always mentioned.

The actual data files as well as the analysis (in Graphpad or Igor pro) were uploaded for your information. All information about the actual data is available in the uploaded Excel and graphpad files. No data points were excluded from the analysis. The zipped source data files contain, apart from the data itself, a summary table with the number of tests that were performed in different preparations dissected from each of the two intestinal regions from different animals.

An extra schematic with an overview of what data were extracted from each type of experiment (single stimulation for the description of number of responders and average amplitude; stimulation pairs from multiple tissues and animals for the ratio’s and ratio histograms).

**Statistical reporting**

- Statistical analysis methods should be described and justified
- Raw data should be presented in figures whenever informative to do so (typically when N per group is less than 10)
- For each experiment, you should identify the statistical tests used, exact values of N, definitions of center, methods of multiple test correction, and dispersion and precision measures (e.g., mean, median, SD, SEM, confidence intervals; and, for the major substantive results, a measure of effect size (e.g., Pearson's r, Cohen's d)
- Report exact p-values wherever possible alongside the summary statistics and 95% confidence intervals. These should be reported for all key questions and not only when the p-value is less than 0.05.

Please outline where this information can be found within the submission (e.g., sections or figure legends), or explain why this information doesn’t apply to your submission:

Statistical analysis is described in the subparagraph of the Materials and Methods (Page 7, line 24-29). Generally the data were presented as averages +/- standard errors and statistical tests used were Student’s t-test, unless mentioned otherwise. e.g. for instance in the case of histogram comparisons, 2-way ANOVA with Bonferroni posthoc tests were used and in the comparison of the proportion of oral and aboral projections of traced neurons, we used a Chi^^^2 test. The actual p-values were mentioned up to 3 decimal digits, unless they were not specified by the statistical program (Graphpad) used.

All this information is also mentioned in the methods section under the heading “Data Analysis”.

All raw data, further analysis details and p-values are given in the text of figure legend for each of the different experiments.

A zipped file of all excel and Graphpad data analysis for each of the graphs in Fig. 1-7 and Suppl Fig. 1 has been uploaded.

(For large datasets, or papers with a very large number of statistical tests, you may upload a single table file with tests, Ns, etc., with reference to sections in the manuscript.)

**Group allocation**

- Indicate how samples were allocated into experimental groups (in the case of clinical studies, please specify allocation to treatment method); if randomization was used, please also state if restricted randomization was applied
- Indicate if masking was used during group allocation, data collection and/or data analysis

Please outline where this information can be found within the submission (e.g., sections or figure legends), or explain why this information doesn’t apply to your submission:

The details about the experimental animals that were used for video recordings, Calcium imaging, immunohistochemistry and viral injections are given in the text. Since no formal comparison between drug or disease conditions were made, there was no allocation into experimental groups.

With respect to the drug experiments, information about the repeats in control solution and/or in drugs is given in the paragraph “calcium imaging” in the materials and methods section.

**Additional data files (“source data”)**

- We encourage you to upload relevant additional data files, such as numerical data that are represented as a graph in a figure, or as a summary table
- Where provided, these should be in the most useful format, and they can be uploaded as “Source data” files linked to a main figure or table
- Include model definition files including the full list of parameters used
- Include code used for data analysis (e.g., R, MatLab)
- Avoid stating that data files are “available upon request”

Please indicate the figures or tables for which source data files have been provided:

A zipped file of all excel and Graphpad data files for each of the graphs in Fig. 1-7 and Suppl Fig. 1 has been uploaded.

Igor pro code used to generate the spatial graphs and the analysis of the Ca image stacks has been uploaded as well.
